# Sarcoptic mange breaks up bottom-up regulation of body condition in a large herbivore population

**DOI:** 10.1186/s13071-015-1188-4

**Published:** 2015-11-06

**Authors:** João Carvalho, José E. Granados, Jorge R. López-Olvera, Francisco Javier Cano-Manuel, Jesús M. Pérez, Paulino Fandos, Ramón C. Soriguer, Roser Velarde, Carlos Fonseca, Arian Ráez, José Espinosa, Nathalie Pettorelli, Emmanuel Serrano

**Affiliations:** Centro de Estudos do Ambiente e do Mar (CESAM), Departamento de Biologia, Universidade de Aveiro, Campus Universitário de Santiago, 3810-193 Aveiro, Portugal; Servei d’Ecopatologia de Fauna Salvatge (SEFaS), Departament de Medicina i Cirurgia Animals, Universitat Autònoma de Barcelona, E-08193, Bellaterra, Barcelona, Spain; Espacio Natural Sierra Nevada, Carretera Antigua de Sierra Nevada, Km 7, E-18071, Pinos Genil, Granada Spain; Departamento de Biología Animal, Biología Vegetal y Ecología, Universidad de Jaén, Campus Las Lagunillas, s.n., E-23071, Jaén, Spain; Agencia de Medio Ambiente y Agua, Isla de la Cartuja, E-41092, Sevilla, Spain; Estación Biológica de Doñana (CSIC), Av. Américo Vespucio, s.n., E-41092, Sevilla, Spain; Institute of Zoology, Zoological Society of London, Regent’s Park, London, NW1 4RY UK

**Keywords:** *Capra pyrenaica*, Host-parasite relationships, Iberian Ibex, Density-dependence, Remote sensing, *Sarcoptes scabiei*

## Abstract

**Background:**

Both parasitic load and resource availability can impact individual fitness, yet little is known about the interplay between these parameters in shaping body condition, a key determinant of fitness in wild mammals inhabiting seasonal environments.

**Methods:**

Using partial least square regressions (PLSR), we explored how temporal variation in climatic conditions, vegetation dynamics and sarcoptic mange (*Sarcoptes scabiei*) severity impacted body condition of 473 Iberian ibexes (*Capra pyrenaica*) harvested between 1995 and 2008 in the highly seasonal Alpine ecosystem of Sierra Nevada Natural Space (SNNS), southern Spain.

**Results:**

Bottom-up regulation was found to only occur in healthy ibexes; the condition of infected ibexes was independent of primary productivity and snow cover. No link between ibex abundance and ibex body condition could be established when only considering infected individuals.

**Conclusions:**

The pernicious effects of mange on Iberian ibexes overcome the benefits of favorable environmental conditions. Even though the increase in primary production exerts a positive effect on the body condition of healthy ibexes, the scabietic individuals do not derive any advantage from increased resource availability. Further applied research coupled with continuous sanitary surveillance are needed to address remaining knowledge gaps associated with the transmission dynamics and management of sarcoptic mange in free-living populations.

## Background

Body condition, i.e. energetic state and fat stores of an animal, is a major determinant of individual performance in most vertebrate species [1], including ungulates [2]. In particular, the increase of body condition in anticipation of food shortages is one of the most common mechanisms displayed by herbivores to prevent starvation in highly seasonal environments [3]. Parasites typically have a deleterious effect on body condition, mainly because infected hosts try to reduce the intensity or length of infestation by allocating resources in the activation of the immune response; they may also try to alleviate the damages caused by the infestation by investing energy in tissue repair and detoxification [4]. Because of this, one can expect the energetic costs of infestation are more pronounced in periods of food shortage [5].

Sarcoptic mange caused by the mite *Sarcoptes scabiei* is an excellent study model to evaluate how parasites may interfere with the bottom-up regulation of body condition in wild mammals inhabiting highly seasonal ecosystems. This mite is responsible for severe epizootic disease outbreaks in a broad range of mammals, sometimes causing increases in mortality rates. Infected animals typically suffer from dramatic structural and functional changes in the skin, becoming listless, dehydrated, emaciated and eventually dying from the infestation [6]. Notwithstanding the increasing knowledge about the immune response [7] and pathology [8] of sarcoptic mange, little is known about the relationship between environmental conditions, mange severity and body condition. This topic is of particular importance for the management of the disease, as hosts facing hard environmental conditions could experience increased mange severity. The energetic demands on the host, coupled with the induced physiological changes, make this parasitic disease particularly worrying for free-living populations inhabiting such environments.

### Sarcoptic mange in the Iberian ibex

Iberian ibex (*Capra pyrenaica* Schinz, 1838) is a medium-sized endemic mountain ungulate commonly affected by sarcoptic mange [9]. Mange outbreaks can result in devastating short-term mortality of ibexes, as happened in the *Sierras de Cazorla, Segura y Las Villas* Natural Park, southern Spain, in the late 80s. There, approximately 95 % of ibexes were killed by the parasite [9]. Since 1992, sarcoptic mange has become endemic in the ibex population of the *Sierra Nevada* Natural Space (SNNS, hereafter). One particularity of this extreme Alpine ecosystem is the strong seasonal climatic variation found in the area, with snow cover present for six months of the year. SNNS ibex are adapted to such contrasted conditions and do display an income breeder strategy, i.e. increasing adipose tissues during the summer in anticipation of food deprivation during winter times [10].

Previous studies have addressed the consequences of mange infestation in SNNS Iberian ibex population: e.g. immune response to first and second exposures [11], effects on individual growth [12], reproductive allocation [13] and seasonal variation of hematology and biochemistry among scabietic ibexes [14]. To date, however, no information exists on how climatic conditions, vegetation dynamics and mange severity impact the body condition of these free-ranging ibexes. Although body weight reduction is detectable at early stages of infestation [14], 80 % of ibexes recover totally from mange [15]. Thus, we can hypothesize that aside from the individual factors that shape resistance to *Sarcoptes scabiei* infestation, the effects of sarcoptic mange on ibex body condition will be influenced by the local environmental conditions (e.g. food availability, winter harshness) experienced during infestation.

Taking advantage of thirteen years (1995–2008) of data on sarcoptic mange monitoring in the Iberian ibex population of SNNS, we explored whether disease severity interacted with primary productivity, snow cover and ibex abundance in determining body condition. The strength of these interactions was assessed during two periods of contrasted vegetation dynamics and snow cover: green (March – October) and dormant period (November – February). We hypothesized that the deleterious effects of mange on ibex body condition will be compensated by favorable environmental conditions, e.g. high primary productivity, little snow cover and low population abundance. To the best of our knowledge, this study provides the first explicit assessment of how a parasitic disease shapes bottom-up processes in a large mammal.

## Methods

### Ethics statement

This study complies with the Spanish and the Andalusian laws regarding bioethics and animal welfare. The *Sierra Nevada* National Park approved this study.

### Study area

**Fig. 1:**
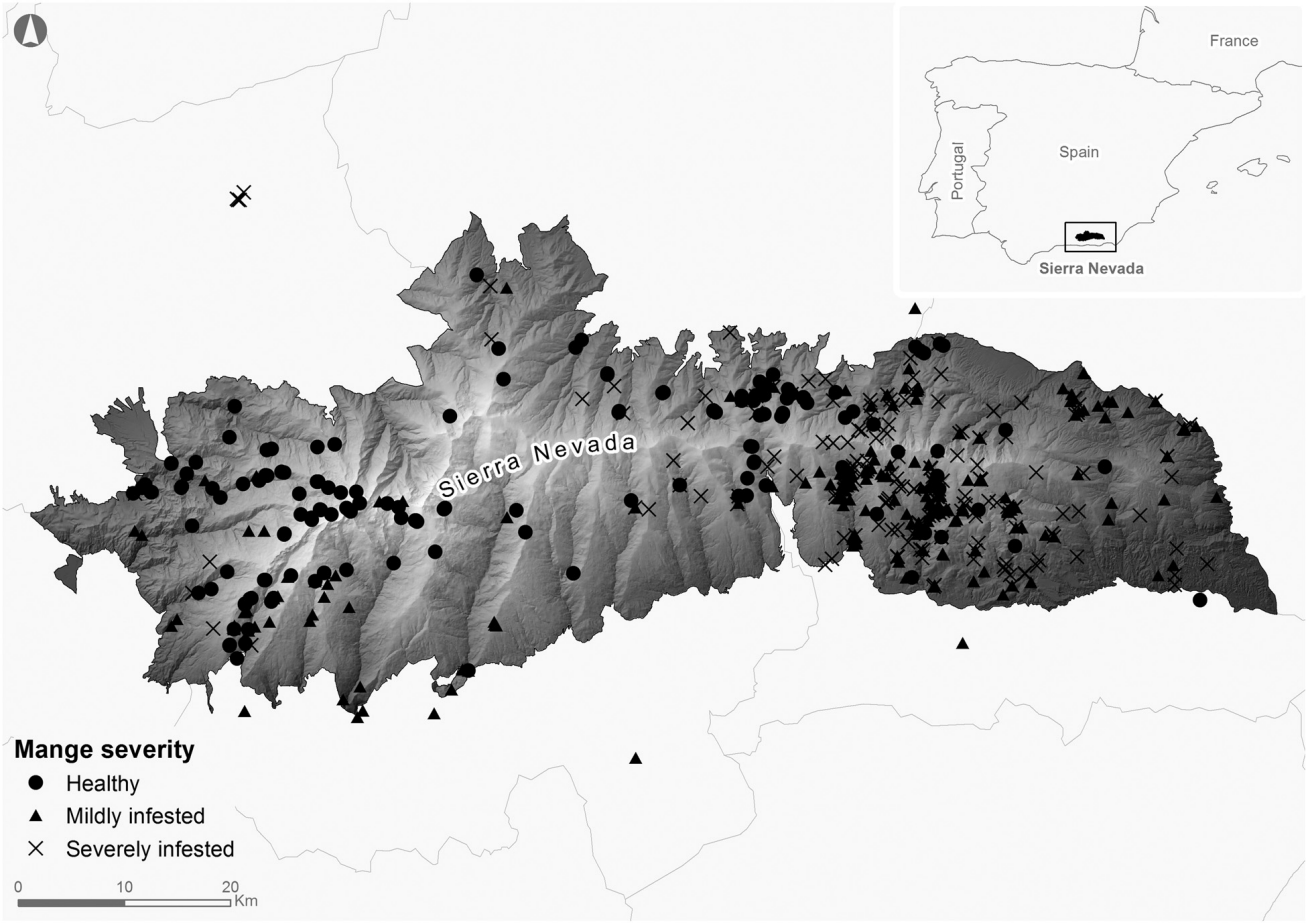
Location of the study area, the Sierra Nevada Natural Space. The spatial distribution of shot-harvested animals and the degree of mange severity (healthy, mildly and severely infested) are also showed

The SNNS covers an area of approximately 2.000 km^2^ and is characterized by a heterogeneous orography, with an altitudinal range between 860 and 3.482 m.a.s.l (Fig. 1). According to the Köppen–Geiger classification system, the SNNS experiences a Mediterranean Subartic climate [16]. Annual average precipitation is about 600 mm [17]. Minimum and maximum average monthly temperatures vary between −5 °C in February and 17 °C in July, with pronounced summer drought. The average annual temperature decreases from 12 to 16 °C below 1.500 m to 0 °C above 3.000 m. Snow generally covers a significant part of the study area between December and May; vegetation growth mainly occurs between June and August. SNNP encompasses the largest and best-known population of Iberian ibex in Andalusia [9].

### Vegetation greenness and snow cover data

**Fig. 2:**
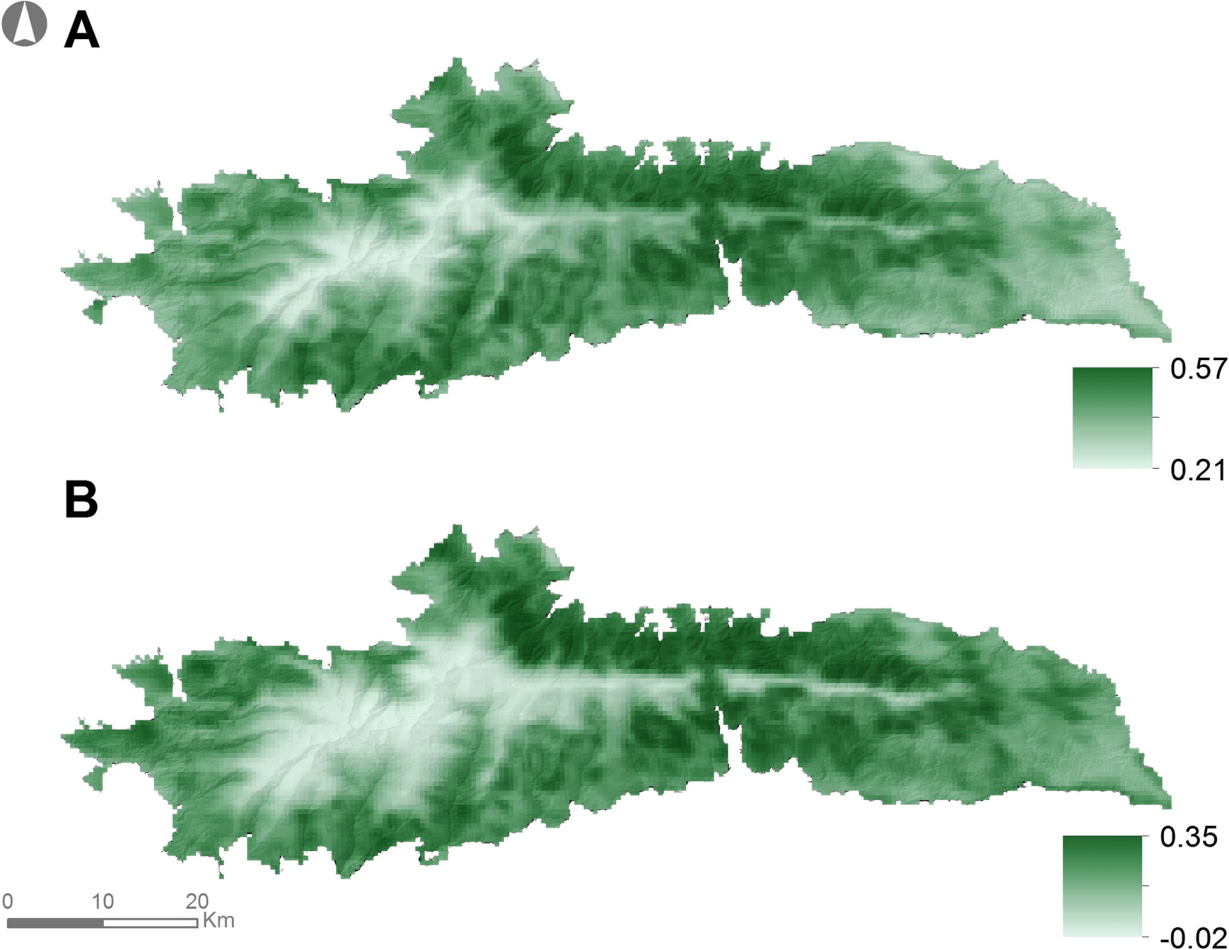
Spatial variability of NDVI during the (**a**) green (March – October) and (**b**) dormant (November – February) periods in SNNS. Pixel values in each map correspond to the pixel average for the relevant season for the period 2000–2008

Two environmental variables were considered while assessing the role of bottom-up processes on the body condition of ibex: the normalized difference vegetation index (NDVI, Fig. 2) used as a proxy of vegetation productivity, and snow cover [18, 19]. The former information was extracted from the MODIS repository (Moderate Resolution Imaging Spectroradiometer; http://modis.gsfc.nasa.gov) at a spatial resolution of 500 m and bi-monthly temporal resolution. Snow cover (percentage of surface covered by snow) was retrieved from the *Observatorio Cambio Global* - *Sierra Nevada* website (http://obsnev.es/linaria.html). Only NDVI values associated with shrubs and herbaceous layers were considered, since ibex primarily feed in areas encompassed by these landcover types in SNNS [20]. Mean monthly NDVI and snow cover values were computed for each year.

### Ibex data

We used block counts to estimate ibex abundance during the study period. In brief, a set of line transects were systematically placed in order to provide an equal coverage of habitats that occur in the study area. Thanks to this approach, the bias related to the systematic prospection of suitable areas was reduced. The survey was conducted every month by a fixed number of teams. Ibexes were observed by means of 8 × 40 binoculars and 20–60 × 65 spotting scopes. For modelling purposes, counts were expressed as the number of ibexes recorded in a given itinerary on a seasonal basis.

A total of 243 male and 230 female Iberian ibexes older than two years were shot between 1995 and 2008, in the context of a mange control program carried out in the SNNS during this period. Sex was determined by visual inspection and the age in years was determined from horn-segment counts [21].

Mange severity was visually assessed using three categories, based on the percentage of skin surface affected by mites [22]: healthy = ibexes without skin lesions, mildly infested = skin surface affected ≤ 50 % and severely infested when skin surface affected > 50 %. Animals were weighed to the nearest 0.1 kg; the kidneys were removed and transported to the laboratory in a cold box at 4 °C. Kidney fat reserves were assessed following Serrano et al. [10] recommendations. Both fat-free kidney mass (KM) and associated peripheral fat (KF) are positively correlated to nutritional status and body condition of a wide range of mammals including Iberian ibex [23]. Therefore, we used the residuals from the linear regression between KM and KF as a proxy of body condition.

### Statistical analysis

Partial least square regressions (PLSR) were used to assess the influence of disease severity, resource dynamics, intra-specific competition and snow cover on body condition. Carrascal et al. [24] defined the PLSR method as an extension of multiple linear regressions in which a response variable (body condition) is modelled through the analysis of linear combinations among predictors (NDVI, snow cover and ibex abundance). This technique is distribution-free and well suited to handle multicollinearity issues, preventing possible misinterpretations of regression coefficients [25]. A PLSR model was developed for each combination of time period (green and dormant period) and mange severity (healthy, mildly and severely infested). Results are presented and interpreted under the assumption that body condition of severely infected ibexes is lower than in healthy animals and of those in early stages of infestation ([26]). The sex of Iberian ibexes was excluded from the analysis because the lack of sex-biased effect of mange on ibexes’ condition ([26]). To minimize the potential effects of body growth on body condition, only individuals close to the final body size were retained in our analysis (≥3 years old [21, 27]). The significance of PLSR models was assessed through the Stone-Geisser Q^2^ test, a cross-validation redundancy measure created to evaluate the predictive significance of the exogenous variables. Test values greater than 0.0975 indicate that the exogenous variables are statistically significant for the response, whereas values below this threshold reveal no significance. The R^2^ was performed to measure the explanatory performance of models developed. Radar plots were used to explore the correlations between the variables and the first two axes associated to the first two components. All calculations were performed in R (version 3.2.0, R Development Core Team 2013). The package “plspm” was used to develop and visualize the PLSR outputs [28].

## Results

Information on the body condition of 473 Iberian ibex was considered in our analyses: 43 (32 males and 11 females) of these individuals were classified as healthy, 217 (92 males and 125 females) as mildly infested and 213 (119 males and 94 females) as severely infested.

**Fig. 3:**
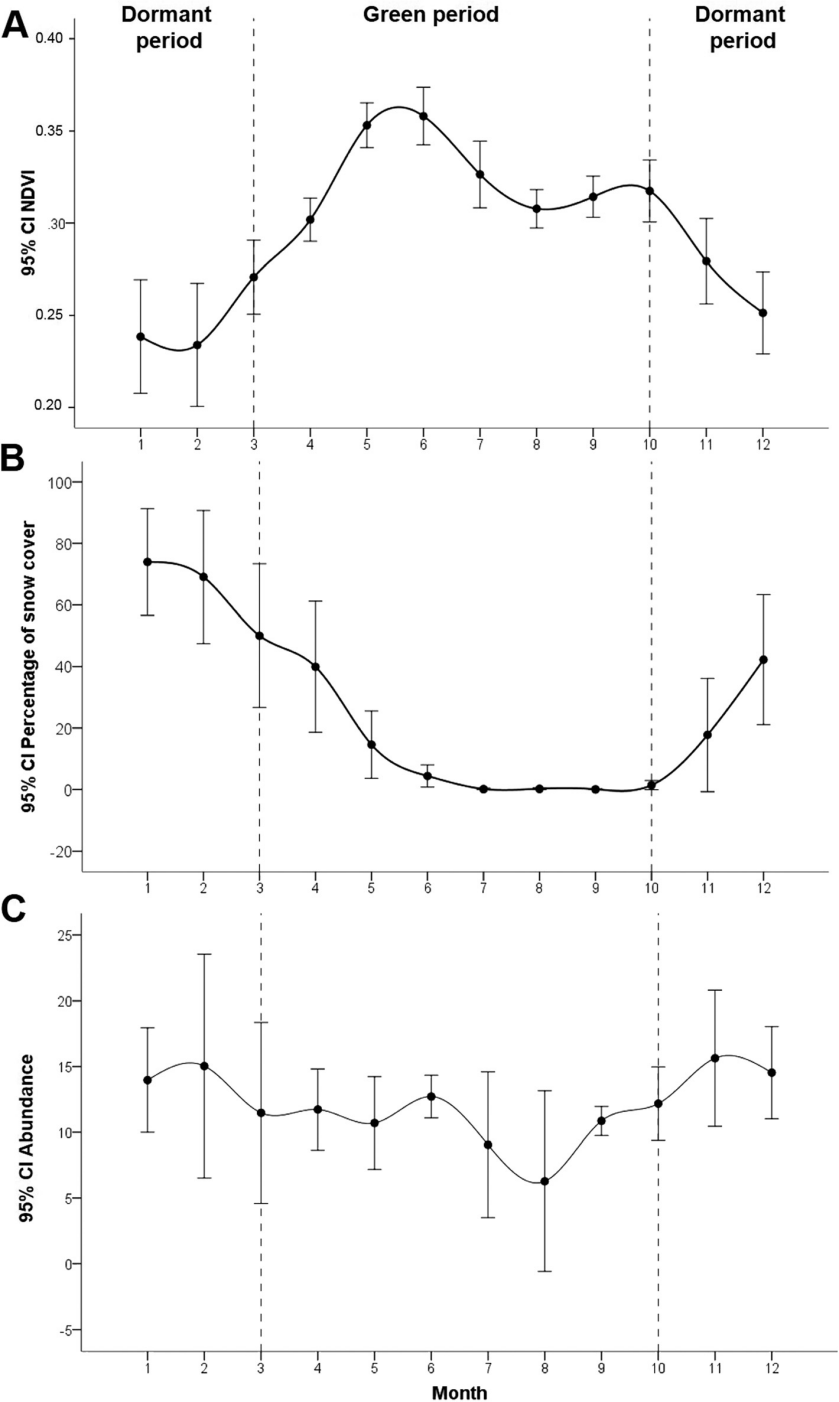
Intrannual variations of NDVI (**a**), snow cover (**b**) and number of ibexes observed (**c**) in SNNS. The error bars represent the inter-annual (1995–2008) fluctuations of variables values

The monthly variations in the vegetation productivity, snow cover and ibex counts are shown in the Fig. 3. As would be expected, vegetation productivity and snow cover were characterized by opposite trends. The number of Iberian ibexes recorded a slight increase during the dormant period comprising the winter and the early spring.

**Fig. 4:**
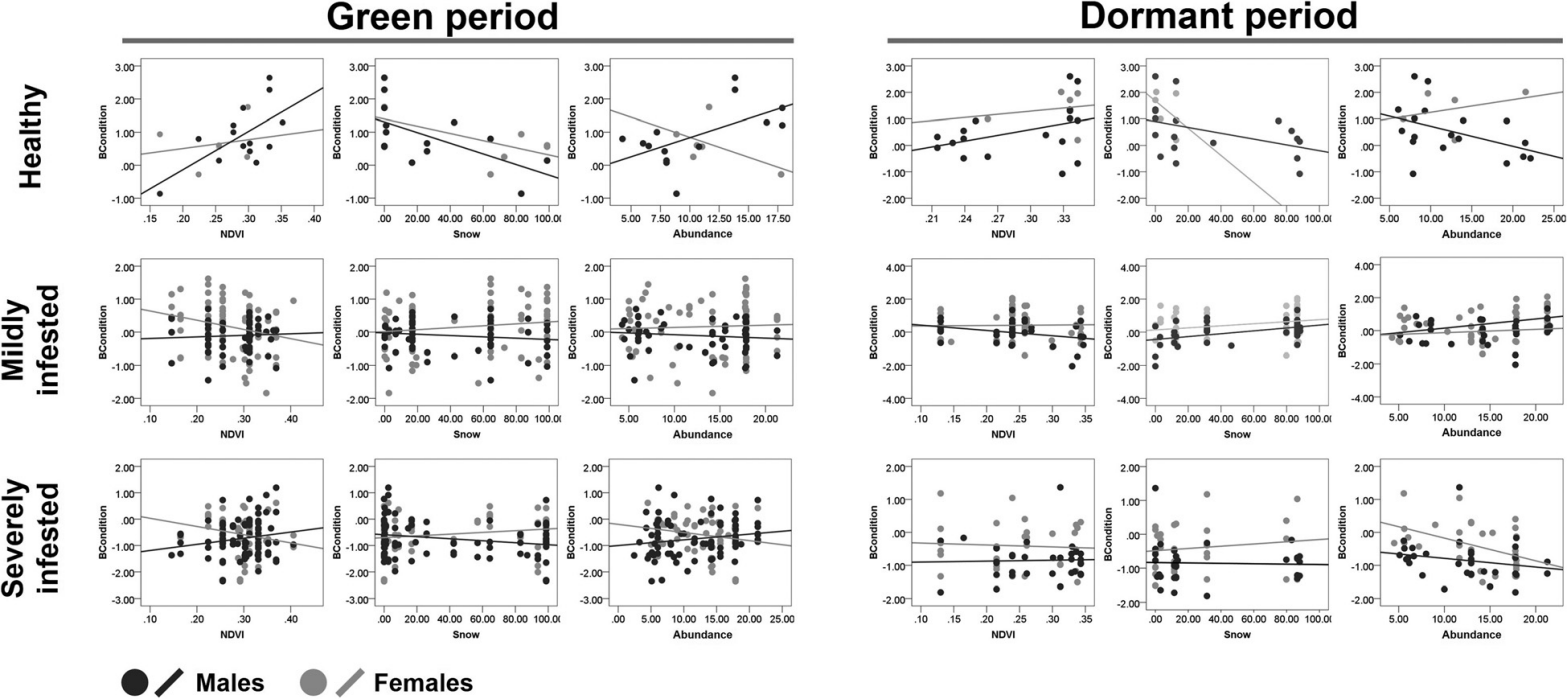
Relation between body condition (males and females) and the three environmental predictors in healthy, mildly and severely infested animals across two time periods of contrasted vegetation productivity (*green* and *dormant*)

**Fig. 5:**
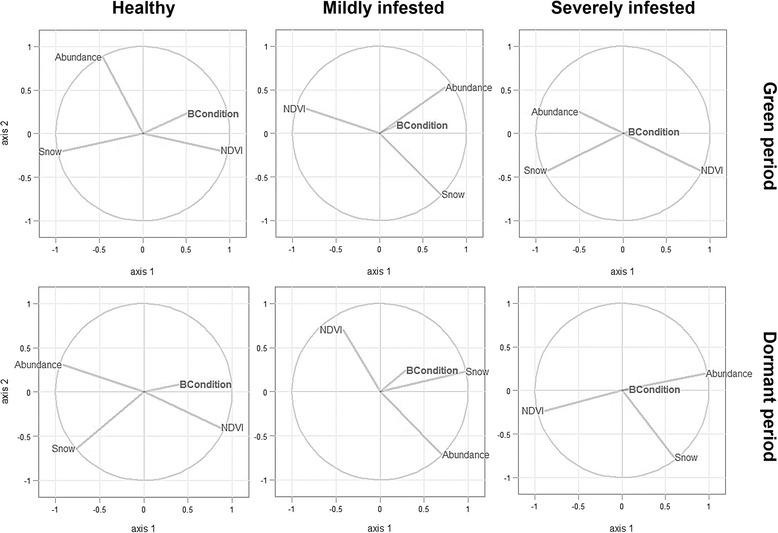
Correlation of the environmental predictors and the response with the first two components. Each segment represents a variable. Longer segments, i.e. closer to the perimeter of the circle, indicate that the corresponding variable is better represented. Segments close to each other represent highly and positively correlated variables. On the other hand, segments in opposite extremes indicate negative correlation. Orthogonal segments mean no correlation among predictors

Summaries of PLSR analyses for each combination of time period and mange severity are presented in the Tables 1 and 2. Through Stone-Geyser’s Q^2^ test we identified that bottom-up regulations of ibex condition only occurred in healthy ibexes (Q^2^ > 0.0975, Table 1). For healthy individuals, the considered environmental predictors as well as population abundance explained 25.14 % and 16.86 % of the observed variability in ibex body condition in the green and dormant period, respectively. Body condition of healthy animals was positively correlated with vegetation productivity (as indexed by average NDVI) and was negatively influenced by snow cover (Fig. 4). Primary productivity (Load = 0.68, w = 0.62) and snow cover (Load = −0.71, w = −0.77) were the main drivers of body condition of healthy animals in the green period whereas the ibexes abundance had greater relevance during the dormant season (Load = −0.62, w = −0.57) (Table 2, Fig. 5). None of the environmental and population factors analyzed influenced body condition of diseased ibexes, either at the mild or severe mange stages of infestation (Table 1). Thus, neither higher primary productivity nor winter harshness and population abundance influenced the impact of mange on ibex body condition.

**Table 1:** R-squared and Stone-Geyser’s *Q* ^*2*^ test values for the partial least squares regression (PLSR) analysis. Each model results from the combination between the two time-periods of contrasted vegetation productivity, and mange severity categories. A component is considered significant if *Q* ^*2*^ ≥ 0.0975

Period	Severity	*R* ^*2*^ (%)	*Q* ^*2*^
Green	Healthy	25.14	0.14
	Mildly infested	3.37	0.01
	Severely infested	0.2	−0.02
Dormant	Healthy	16.86	0.11
	Mildly infested	8.43	0.06
	Severely infested	0.01	−0.02

**Table 2:** Predictor weights and loads for the first component of partial least squares regression (PLSR) analysis. The contribution of each environmental predictor to the PLSR’s axis X is represented by the predictor weights

Period	Severity	Predictors	Loads	Weights
Green	Healthy	NDVI	0.68	0.62
		Snow	−0.71	−0.77
		Abundance	−0.35	−0.04
	Mildly infested	NDVI	−0.66	−0.66
		Snow	0.55	0.25
		Abundance	0.60	0.70
	Severely infested	NDVI	0.67	0.59
		Snow	−0.65	0.75
		Abundance	−0.38	0.38
Dormant	Healthy	NDVI	0.59	0.55
		Snow	−0.52	−0.62
		Abundance	−0.62	−0.57
	Mildly infested	NDVI	−0.39	−0.29
		Snow	0.91	0.96
		Abundance	0.66	0.02
	Severely infested	NDVI	−0.62	−0.61
		Snow	0.42	0.36
		Abundance	0.67	0.70

## Discussion and conclusions

This study provides the first known quantification of the impact of sarcoptic mange on the bottom-up regulation of body condition in a mountain ungulate. Our analyses led to three main results: i) an increase in primary productivity clearly triggers an increase in body condition in healthy ibexes, ii) sarcoptic mange can disrupt the link between environmental conditions and body condition, and iii) body condition is independent from ibex abundance parameter in scabietic ibexes.

The absence of a bottom-up regulation of body condition in scabietic individuals may be related to the physiopathology of this parasitic disease, which is characterized by emaciation, muscle mass losses and anemia [6, 29]. Even at the early/mild stages of infestation, mange can result in anemia (i.e. RBC, Hb, hematocrit reduction), accelerating the net catabolism of the body protein storage in ibex (i.e. increased blood urea and decreased creatine concentration) [14]. Such pathological and physiological changes have been reported in several other species inhabiting a wide range of environments (coyotes (*Canis latrans*), [30]; rabbits (*Oryctolagus cuniculus*), [31]; red foxes (*Vulpes vulpes*), [32]; wombats (*Vombatus ursinus*), [33]). For instance, Skerrat et al. [33] showed that wombats were using their body stores to cope with the energetic costs of sarcoptic mange. Likewise, Arlian et al. [31] concluded that the energy demand in rabbits is driven by mange severity i.e. severity increases the energetic costs to handle weight loss. These costs probably hinder the restoration of energy reserves in scabietic ibexes with severe hyperkeratotic lesions. However, in Iberian ibex the negative effects of sarcoptic mange on the capability to restore reserves was also present in the mildly stages of the disease. Indeed, sarcoptic mange exerts a negative and seasonal effect on body weight of infested Iberian ibexes [14], compromising the daily weight gain.

When overabundance coincides with limited food availability, the ability of the hosts to cope with infestations can be compromised [34]. Here, we failed to detect density-dependence in the bottom-up regulation of body condition in scabietic ibexes (see also Fernández-Morán et al. [35] who reported the absence of correlation between host population density and mange prevalence in Cantabrian chamois (*Rupicapra pyrenaica parva*). It has been suggested that host’s abundance predisposes the population to mange infestation [36, 37], since host-to-host transmission is favored in crowded host populations [15, 38]. In fact, aggregation improves disease maintenance [39] and host susceptibility increase when high host densities coincide with limited food availability [5]. Despite all these evidences, once infected, food shortage due to intra-specific competition appears to have no effect on the body condition of infected individuals.

In healthy ibexes, the negative relationship between population abundance and body condition is less pronounced in the green period than in the dormant season. This result could be explained by the habitat use of Iberian ibex. As with other closely related *Caprinae* species [40], ibexes can be easily seen around alpine meadows and pastures looking for sites free from snow and with fresh vegetation. However, during the dormant period the strong and negative relationship between population abundance and body condition can be explained by the fact that available resources are probably not sufficient to cover ibex’ energetic needs. Such a period also coincides with a reduction of forage intake and the typical increase of energy expenditure due to rut [41]. Therefore, whereas food shortage in the dormant period would act as a classical population bottom-up regulator, higher food availability during the high production green period would allow the Iberian ibex population to thrive.

Here, we have shown how scabietic ibexes do not take advantage of increases in resource availability, which is a drawback to the implementation of management practices, at least in the short term. A very recent work [15] underlined the ability of ibexes to survive from mange infestation, which opens a new window to disease management. Further research should be focused on the effects of habitat management on the progression of mange and the survival of scabietic individuals.
